# DREAMSeq: An Improved Method for Analyzing Differentially Expressed Genes in RNA-seq Data

**DOI:** 10.3389/fgene.2018.00588

**Published:** 2018-11-30

**Authors:** Zhihua Gao, Zhiying Zhao, Wenqiang Tang

**Affiliations:** ^1^Ministry of Education Key Laboratory of Molecular and Cellular Biology, Hebei Key Laboratory of Molecular and Cellular Biology, Hebei Collaboration Innovation Center for Cell Signaling, College of Life Sciences, Hebei Normal University, Shijiazhuang, China; ^2^College of Biological Science and Engineering, Hebei University of Economics and Business, Shijiazhuang, China

**Keywords:** RNA-seq, DREAMSeq, equidispersion, overdispersion, underdispersion, double Poisson model, negative binomial model

## Abstract

RNA sequencing (RNA-seq) has become a widely used technology for analyzing global gene-expression changes during certain biological processes. It is generally acknowledged that RNA-seq data displays equidispersion and overdispersion characteristics; therefore, most RNA-seq analysis methods were developed based on a negative binomial model capable of capturing both equidispersed and overdispersed data. In this study, we reported that in addition to equidispersion and overdispersion, RNA-seq data also displays underdispersion characteristics that cannot be adequately captured by general RNA-seq analysis methods. Based on a double Poisson model capable of capturing all data characteristics, we developed a new RNA-seq analysis method (DREAMSeq). Comparison of DREAMSeq with five other frequently used RNA-seq analysis methods using simulated datasets showed that its performance was comparable to or exceeded that of other methods in terms of type I error rate, statistical power, receiver operating characteristics (ROC) curve, area under the ROC curve, precision-recall curve, and the ability to detect the number of differentially expressed genes, especially in situations involving underdispersion. These results were validated by quantitative real-time polymerase chain reaction using a real Foxtail dataset. Our findings demonstrated DREAMSeq as a reliable, robust, and powerful new method for RNA-seq data mining. The DREAMSeq R package is available at http://tanglab.hebtu.edu.cn/tanglab/Home/DREAMSeq.

## Introduction

With the development of next-generation sequencing technology, RNA sequencing (RNA-seq) has become a routine and powerful method for evaluating global dynamic changes in gene expression during certain biological processes. Compared with microarray technologies, RNA-seq technologies have several advantages, including a wider measurable range of expression levels, higher throughput, less noise, more information for detecting allele-specific expression, and a higher capability to detect novel promoters and alternative gene-splicing isoforms (Marioni et al., 2008; Mortazavi et al., 2008; Sultan et al., 2008; Wang et al., 2009, 2010b; Oshlack et al., 2010). Therefore, developing powerful, reliable, and unbiased RNA-seq data-mining methods would facilitate the use of RNA-seq to explore basic biological questions in this era of big data.

Typically, RNA-seq experimental procedures can be divided into six steps: (1) sequencing the RNA samples to obtain raw reads, (2) filtering out low-quality reads, (3) mapping the high-quality reads to a reference genome or transcriptome, (4) summarizing the read counts for each gene, (5) detecting differentially expressed genes (DEGs), and (6) performing systems biology analysis [e.g., cluster analysis, principal components analysis (PCA), gene ontology (GO) analysis, and pathway enrichment analysis] (Oshlack et al., 2010). Of these steps, identifying DEGs across treatments/conditions is the key task and often the primary goal of RNA-seq data analysis. There are numerous statistical methods focusing directly on read-count data for DEG identification, with these classified into two categories: (1) parametric methods that rely on assumptions about discrete probability models and include methods based on a Poisson model, such as DEGseq (Wang et al., 2010a) and TSPM (Auer and Doerge, 2011), methods based on a negative binomial (NB) model, such as edgeR (Robinson et al., 2010), DESeq (Anders and Huber, 2010), baySeq (Hardcastle and Kelly, 2010), NBPSeq (Di et al., 2011), EBSeq (Leng et al., 2013), ShrinkSeq (Van De Wiel et al., 2013), and DESeq2 (Love et al., 2014), methods based on a beta-binomial model, such as BBSeq (Zhou et al., 2011), methods based on a multivariate Poisson log-normal (LN) model, such as PLNseq (Zhang et al., 2015), and methods based on a generalized Poisson (GP) model, such as GPseq (Srivastava and Chen, 2010) and deGPS (Chu et al., 2015); and (2) non-parametric methods, such as NOISeq (Tarazona et al., 2011) and SAMseq (Li and Tibshirani, 2013), that do not assume any particular model.

Among count-based RNA-seq data-analysis methods, non-parametric methods were developed based on large-sample asymptotic theory and exhibit statistical power sufficient to detect DEGs only when the number of replicates per treatment condition is ≥5 (Tarazona et al., 2011; Seyednasrollah et al., 2013; Soneson and Delorenzi, 2013). However, due to the high cost of RNA-seq, the general sample size in a typical RNA-seq experiment is < 5 replicates, which limits the application of non-parametric methods in RNA-seq data mining. Therefore, the most popular RNA-seq data-analysis methods are parametric methods based on Poisson and NB models. In early RNA-seq studies where only technical replicates were used, the traditional Poisson model was highly capable of fitting read-count data characterized by equidispersion (i.e., the variance is equal to the mean) (Marioni et al., 2008; Bullard et al., 2010). However, when biological replicates are available, read-count data often exhibits more variability than the Poisson model expects, which limits the use of a Poisson model for analyzing RNA-seq data (Anders and Huber, 2010). Fortunately, the NB model, as a Gamma-Poisson mixture, can address the overdispersion issue (i.e., when the variance is larger than the mean), as well as capture equidispersion (Anders and Huber, 2010). Additionally, recent studies reported that some RNA-seq data demonstrates characteristics of underdispersion (i.e., the variance is smaller than the mean), which might be caused by RNA-seq coverage, as well as zero-inflation, cluster, or low expression level of the count data, and could lead to underestimation of DEGs (Famoye, 1993; Srivastava and Chen, 2010; Rau et al., 2011; Mi et al., 2015; Choo-Wosoba et al., 2016; Low et al., 2017). However, neither a traditional Poisson model nor the NB model works well at mining underdispersed data.

The GP model is a generalization of the Poisson model with an additional parameter. This method can process data characterized by underdispersion and non-underdispersion (equidispersion and overdispersion) (LuValle, 1990), but can only capture certain levels of dispersion, because the model is truncated under certain conditions regarding its bounded dispersion parameter (Famoye, 1993). For example, the program deGPS employs the GP model to fit read-count data characterized by non-underdispersion (Chu et al., 2015), whereas GPseq uses this model to consider potential positional bias during DEG analysis and handle position-level counts instead of gene-level counts, which is different from other methods (Srivastava and Chen, 2010). Therefore, these methods derived from different discrete models can potentially perform poorly at fitting underdispersed count data due to the restrictions associated with the inherent properties in the models.

In this study, we described a mixed Poisson model called double Poisson (DP), which offers the advantage of flexibility in fitting a wide range of data exhibiting underdispersion and non-underdispersion using only two parameters (Efron, 1986). Based on this model, we developed a novel differential relative expression-analysis method for RNA-seq data mining (DREAMSeq). Because the results of differential gene-expression analysis are dependent upon the discrete model used to fit the RNA-seq data (Consortium, 2010), we also added NB-model functionality to the DREAMSeq pipeline in order to optimize the performance of our method. Therefore, depending on the model used in the pipeline, our method can be divided into three approaches: DREAMSeq.DP (based on the DP model), DREAMSeq.NB (based on the NB model), and DREAMSeq.Mix (based on the mixture of the DP and NB models, with the lower *p*-value between two *p*-values generated based on the DP and NB models chosen as the final *p*-value) in order to fit variable RNA-seq data. In order to evaluate the performance of DREAMSeq, we generated three simulated datasets using three real RNA-seq datasets. Because the DEGs can only be effectively identified when the sample size is ≥3 (Conesa et al., 2016; Lin et al., 2016), to assess DREAMSeq using the most common RNA-seq scenario, we focused on detecting DEGs under small sample sizes (three replicates per condition) and between two groups. Our results indicated that the performance of DREAMSeq at effectively detecting DEGs was comparable to other popular RNA-seq data-analysis methods, including edgeR, DESeq, DESeq2, NBPSeq, and TSPM, in non-underdispersion situations, but outperformed most of the other methods in underdispersion situations. This conclusion was validated by quantitative real-time polymerase chain reaction (qRT-PCR) using a real Foxtail dataset generated in our laboratory. Our findings demonstrated DREAMSeq as a reliable and robust DEG-detection method that provides an additional option in the RNA-seq data-analysis toolbox, especially for underdispersed-data mining.

## Materials and methods

### Models and normalization

In this study, let Y represent the observed count and X the corresponding underlying gene expression (unknown) in an RNA-seq experiment. Let Y_ijk_ and X_ijk_ denote the read count and the true gene expression of gene i from sample j in treatment group k, where i = 1, …, I (the number of genes), j = 1, …, J (the number of replicates; here, J = 3), and k = 1, …, K (the number of groups; here, K = 2), respectively.

#### NB model

We assume that Y follows an NB model with two parameters: the mean, μ, and the dispersion, ϕ. The probability mass function (PMF) of the NB model is given as:

(1) $$\begin{array}{l} P\left(Y=y|\mu ,\phi \right)=\frac{\Gamma \left(y+\phi^{-1}\right)}{y!\Gamma \left(\phi^{-1}\right)}{\left(\frac{1}{1+\mu \phi }\right)}^{\phi^{-1}}{\left(\frac{\mu \phi }{1+\mu \phi }\right)}^{y}. \end{array}$$

The expected value is estimated as:

(2) $$\begin{array}{l} E\left(Y\right)=\mu . \end{array}$$

We parameterize the variance of the NB model according to a previous study (Robinson and Smyth, 2007):

(3) $$\begin{array}{l} {Var\left(Y\right)=\sigma }^{2}=\mu +\mu^{2}\phi , \end{array}$$

where ϕ ≥ 0 and determines the extra variability as compared with the Poisson model. When ϕ > 0, σ^2^ > μ; and when ϕ = 0, σ^2^ = μ; the NB model collapses to the Poisson model, which can be viewed as a special NB model with zero dispersion (Robinson and Smyth, 2007). Therefore, the NB model allows for both overdispersion and equidispersion.

#### DP model

We assume that Y follows a DP model with two parameters: the mean, μ, and the dispersion, θ. The approximate PMF of the DP model is given as:

(4) $$\begin{array}{l} P\left(Y=y|\mu ,\theta \right)=f_{\mu ,\theta }\left(y\right)=\left(\theta^{\frac{1}{2}}e^{-\theta \mu }\right)\left(\frac{e^{-y}y^{y}}{y!}\right){\left(\frac{e\mu }{y}\right)}^{\theta y}. \end{array}$$

The exact DP density is:

(5) $$\begin{array}{l} P\left(Y=y|\mu ,\theta \right)={\tilde{f}}_{\mu ,\theta }\left(y\right)=c\left(\mu ,\theta \right)f_{\mu ,\theta }\left(y\right), \end{array}$$

where the factor c(μ,θ) can be calculated as:

(6) $$\begin{array}{l} \frac{1}{c\left(\mu ,\theta \right)}=\sum_{y=0}^{\infty }f_{\mu ,\theta }\left(y\right)\approx 1+\frac{1-\theta }{12\mu \theta }\left(1+\frac{1}{\mu \theta }\right) \end{array}$$

with *c*(μ, θ) being the normalizing constant nearly equal to 1. The constant *c*(μ, θ) ensures that the density integrates to unity. The expected value and the variance of the DP model in reference to the exact density ${\tilde{f}}_{\mu ,\theta }\left(y\right)$ are estimated as follows:

(7) $$\begin{array}{l} E\left(Y\right)\approx \mu \end{array}$$

and

(8) $$\begin{array}{l} {Var\left(Y\right)=\sigma }^{2}=\frac{\mu }{\theta }, \end{array}$$

respectively, where θ > 0 under RNA-seq data circumstances. The Poisson model is nested in the DP model for θ = 1, indicating that the DP model can fit equidispersed read-count data when θ = 1. Additionally, the DP model allows for both overdispersion (0 < θ < 1) and underdispersion (θ > 1) (Efron, 1986).

#### Normalization

Here, we assume that the expectation of Y_ijk_, μ_ijk_, is the product of X_ijk_ and s_jk_:

(9) $$\begin{array}{l} \mu_{ijk}=X_{ijk}s_{jk}, \end{array}$$

where s_jk_ is the size factor corresponding to sample j in treatment group k, which can be estimated using various existing normalization methods, such as total counts, upper quartile (Bullard et al., 2010), median (Dillies et al., 2012), quantile (Bolstad et al., 2003; Irizarry et al., 2003), trimmed mean of *M*-values (TMM) (Robinson and Oshlack, 2010), DESeq normalization (DESeq) (Anders and Huber, 2010), reads per kilobase per million (RPKM) (Mortazavi et al., 2008), to remove unwanted variation (Risso et al., 2014). Normalization is a process that makes unit-less data comparable among measurements by adjusting for sequencing depth and potentially other technical effects of different samples. Dillies et al. (2012) and Lin et al. (2016) found that TMM and DESeq normalization methods performed much better than the other methods described here. Therefore, the most widely used TMM method was chosen as the default data-normalization method in DREAMSeq and similar to previous studies (Robinson et al., 2010; Kadota et al., 2012; Soneson and Delorenzi, 2013; Sun et al., 2013).

### Dispersion estimations

Estimating the dispersion parameter is a crucial step in DEG detection. Various dispersion-parameter estimation methods, including pseudo-likelihood (Smyth, 2003), quasi-likelihood (Nelder, 2000; Lund et al., 2012), conditional maximum likelihood (CML) (Smyth and Verbyla, 1996), quantile-adjusted CML (Robinson and Smyth, 2008), and shrinkage-estimation methods (Anders and Huber, 2010; Robinson et al., 2010), have been discussed previously. In particular, many Bayesian-based shrinkage-estimation methods, including baySeq, ShrinkSeq, DSS (Wu et al., 2013), and DESeq2, have been developed and are capable of obtaining accurate and robust estimates by sharing information across all genes when the sample size is small (Ji and Liu, 2010). Therefore, we also utilized an empirical Bayesian framework to shrink the dispersion parameter. Our strategy to estimate the dispersion parameter was divided into five steps described as follows.

#### Initial dispersion estimators

We first applied the method-of-moments (MoMs) described by Love et al. (2014) to estimate the initial value of dispersion for each gene. According to previous studies (Anders and Huber, 2010; Robinson et al., 2010), we first use the normalized sample mean, ${\bar{X}}_{ik}$, to estimate the expectation for the i^th^ gene in group k:

(10) $$\begin{array}{l} \mu_{ik}=\frac{1}{J}{\bar{X}}_{ik}\sum_{j}s_{jk}. \end{array}$$

We assume that the dispersions between two groups are the same under small sample sizes. Therefore, we denote *n* = KJ and substitute equation (10) with the following equation:

(11) $$\begin{array}{l} \mu_{i}=\frac{1}{n}{\bar{X}}_{i}\sum_{n}s_{jk}, \end{array}$$

where μ_*i*_ and ${\bar{X}}_{i}$ are the expectation and sample mean, respectively, of the i^th^ gene. We then estimate the variance of the i^th^ gene, $\sigma_{i}^{2}$, by pooling count data from different groups using approaches previously described by Anders and Huber (2010) and Wu et al. (2013). For the NB model, the initial dispersion for the i^th^ gene can be estimated by:

(12) $$\begin{array}{l} \phi_{i}^{init}=\frac{\sigma_{i}^{2}-\mu_{i}}{\mu_{i}^{2}}. \end{array}$$

Note that $\phi_{i}^{init}$ is often artificially assigned with an extremely low positive value (e.g., 1 × 10^−8^ in DESeq) when $\sigma_{i}^{2}<\mu_{i}$, because the NB model cannot fit underdispersed read-count data. A similar conservative strategy was also utilized for underdispersion in a previous study (Schissler et al., 2015). Under this scenario, the initial dispersion can be overestimated, which results in a conservative DEG test (Robinson and Smyth, 2008). By contrast, instead of the NB model, the DP model is capable of handling this kind of data. For the DP model, the initial dispersion for the i^th^ gene can be estimated by:

(13) $$\begin{array}{l} \theta_{i}^{init}=\frac{\mu_{i}}{\sigma_{i}^{2}}. \end{array}$$

#### Gene-wise dispersion estimators

In RNA-seq experiments, there are typically tens of thousands of genes, but only a few replicates per treatment group, which describes the “large p and small n” phenomenon. It is quite difficult to estimate a reliable gene-specific dispersion with the MoMs described in such a scenario. To address this problem, we used maximum likelihood estimate (MLE) methods based on the initial dispersion estimator, $\phi_{i}^{init}$ (or $\theta_{i}^{init}$), to estimate a gene-wise dispersion, $\phi_{i}^{genewise}$ (or $\theta_{i}^{genewise}$), for gene, i. The MLE of the dispersion parameters in the NB and DP models can be obtained by maximizing the log-likelihood summed over all reads between conditions for the i^th^ gene:

(14) $$\begin{array}{l} \phi_{i}^{genewise}=argmax_{\phi }\left(\sum_{n}\log\left(f_{NB}\left(Y_{ijk},\mu_{ik},\phi \right)\right)\right) \end{array}$$

and

(15) $$\begin{array}{l} \theta_{i}^{genewise}=argmax_{\theta }\left(\sum_{n}\log\left(f_{DP}\left(Y_{ijk},\mu_{ik},\theta \right)\right)\right), \end{array}$$

respectively, where $\phi =\phi_{i}^{init}$, $\theta =\theta_{i}^{init}$, and *f*_*NB*_(·) and *f*_*DP*_(·) are the PMF of the NB and DP models, respectively.

#### Common dispersion estimators

It is essential for reliable dispersion estimation that information is shared between genes, especially when few replicates are available (Robinson and Smyth, 2008). The simplest method of sharing information is to assume that the dispersion parameters are common for all genes and then to use the entire dataset to directly calculate a precise common dispersion. However, it is generally not true that each gene has the same dispersion in practice (Robinson and Smyth, 2007). Consequently, we should seek a more general common dispersion-estimation approach that compromises between entirely individual gene-wise dispersions and an entirely shared common dispersion. Here, we assumed that the dispersions are common across all genes having similar expression strengths, suggesting that if the means for some genes are similar, the dispersions (or variances) for these genes are also similar. We adopted a similar locally weighted regression as that for voom (Law et al., 2014) in order to obtain the common dispersion estimators ($\phi_{i}^{common}$ for the NB model or $\theta_{i}^{common}$ for the DP model) for the i^th^ gene by regressing the gene-wise dispersion estimators, $\phi_{i}^{genewise}$ (or $\theta_{i}^{genewise}$), onto the means, μ_*i*_, of the normalized read counts. This is similar to the data-driven parameter estimation used by DESeq through the smooth function by modeling the observed mean-variance (or mean-dispersion) relationship for the genes in the read-count data (Anders and Huber, 2010).

#### Shrinkage-dispersion estimators

Shrinkage estimation can effectively improve statistical tests for differential gene expression in the case of a small number of samples (Cui et al., 2005). As mentioned previously, in order to obtain a more accurate and robust estimate, an empirical Bayes (EB) approach has been used to shrink gene-wise dispersions toward common dispersions, which could effectively allow the borrowing of information between genes (Robinson and Smyth, 2007; Robinson et al., 2010). The DSS and DESeq2 methods use an EB approach incorporating shrinkage with an NB model to squeeze the gene-wise dispersion estimates toward an LN prior, where the strength of shrinkage is dependent upon how reliably the individual gene-wise dispersions can be estimated (Wu et al., 2013; Love et al., 2014). Here, we assumed that the gene-wise dispersions, α, followed an LN prior with two parameters: the mean, m_0_, and the standard deviation (SD), τ. The PMF of the LN model is given as:

(16) $$\begin{array}{l} P\left(\alpha |m_{0},\tau \right)=\frac{1}{\alpha \sqrt{2\pi \tau^{2}}}e^{-\frac{{\left(\log\left(\alpha \right)-m_{0}\right)}^{2}}{2\tau^{2}}}, \end{array}$$

where α represents $\phi_{i}^{genewise}$ and $\theta_{i}^{genewise}$ for the NB and DP models, respectively. The two parameters of the LN model are estimated as follows:

(17) $$\begin{array}{l} m_{0}=median\left(log\left(\beta \right)\right) \end{array}$$

and

(18) $$\begin{array}{l} \tau =mad\left(\log\left(\alpha \right)-log\left(\beta \right)\right), \end{array}$$

respectively, where mad represents the median absolute deviation, and β represents $\phi_{i}^{common}$ and $\theta_{i}^{common}$ for the NB and DP models, respectively.

We adopted the same strategy as the DSS and DESeq2 methods to estimate the shrinkage dispersions for the i^th^ gene in the NB and DP models:

(19) $$\begin{array}{l} \phi_{i}^{shrinkage}=argmax_{\phi }\left(\sum_{n}\log\left(f_{NB}\left(Y_{ijk},\mu_{ik},\phi \right)\right)+f_{LN}\left(\phi ,m_{0},\tau \right)\right) \end{array}$$

and

(20) $$\begin{array}{l} \theta_{i}^{shrinkage}=argmax_{\theta }\left(\sum_{n}\log\left(f_{DP}\left(Y_{ijk},\mu_{ik},\theta \right)\right)+f_{LN}\left(\theta ,m_{0},\tau \right)\right) \end{array}$$

respectively, where $\phi =\phi_{i}^{genewise}$, $\theta =\theta_{i}^{genewise}$, and *f*_*NB*_(·), *f*_*DP*_(·), and *f*_*LN*_(·) are the PMF of the NB, DP, and LN models, respectively.

#### Final dispersion estimators

Bias in dispersion estimation has serious effects on the expected false-positive rates (FPRs) in small-sample situations (Robinson and Smyth, 2008). To avoid bias, DESeq by default chooses the maximum value from the two dispersion estimators: the individual dispersion and the fitted dispersion as a final dispersion for the gene (Anders and Huber, 2010). However, DESeq is often overly conservative due to overestimation of the dispersion and results in conservation tests (Robinson and Smyth, 2008; Soneson and Delorenzi, 2013). For this reason, we proposed a compromise approach called “window scan” to obtain the final dispersion estimators in five steps: (1) rank the genes from smallest to largest according to the means of samples across all conditions; (2) open a default 1-count window, where the mean is smallest; (3) based on the relationship between the shrinkage-dispersion estimator and the common-dispersion estimator, all genes in this window are divided into I-type genes (its shrinkage-dispersion estimator ≥ its common dispersion estimator) and II-type gene (its shrinkage dispersion estimator < its common dispersion estimator); (4) estimate the final dispersion of each I-type gene (or II-type gene) by choosing the larger value between its shrinkage-dispersion estimator and the median of the shrinkage-dispersion estimators of all I-type genes (or II-type genes) for the NB model (or choosing the smaller value for the DP model); and (5) shift the window to the larger mean and repeat steps (3,4) until all of the genes are scanned.

### Test statistic and method evaluation

#### Test statistic

For DEGs detected between two treatment groups, we tested the hypotheses of the form H_0_: μ_i, 1_ = μ_i, 2_ for the gene i, where μ_i, 1_ and μ_i, 2_ are the expectations for the i^th^ gene in groups 1 and 2, respectively. The Wald test has been widely applied in many previous studies because of its simplicity and flexibility (Ng and Tang, 2005; Chen et al., 2011; Yu et al., 2017). Similar to DSS and DESeq2, we constructed the Wald test statistic as:

(21) $$\begin{array}{l} W=\frac{\left|\mu_{i,1}-\mu_{i,2}\right|}{\sqrt{\sigma_{i,1}^{2}+\sigma_{i,2}^{2}}}, \end{array}$$

where $\sigma_{i,1}^{2}$ and $\sigma_{i,2}^{2}$ are the variances for the i^th^ gene in groups 1 and 2, respectively, and can be estimated using the final dispersion according to equation (3) in the NB model and equation (8) in the DP model.

#### Method evaluation

All methods analyzed will return nominal *p*-values. In order to obtain a more reliable list of DEGs, the *p*-values were adjusted by the Benjamini-Hochberg (BH) procedure (Benjamini and Hochberg, 1995). We evaluated the type I error rates (i.e., FPRs) and statistical powers (i.e., true-positive rates; TPRs) of different methods with a significance level of 0.05. Additionally, we used a receiver operating characteristic (ROC) curve, the area under the ROC curve (AUC), and a precision-recall curve (PRC) to compare the performances of eight methods in the simulated datasets. It is common for biologists to be interested in detecting genes with fold changes (FCs) estimated according to the ratios of the mean normalized counts between two treatment groups. Therefore, some methods use FC as an indicator of DE, such as DEGseq and AMAP.Seq (Si and Liu, 2013). Here, we defined the genes satisfying either FC < 0.67 or FC > 1.5, and an adjusted *p* < 0.05 as DEGs according to previous studies (Peart et al., 2005; Si and Liu, 2013). This quantitative filter combines the significance level with the FC threshold and might be considered more practical by biologists. Therefore, we also identified DEGs using this filter.

The performances of different methods were further validated by qRT-PCR analysis.

### Datasets

#### Real datasets

We chose three real datasets to represent different characteristics of RNA-seq data. The Pickrell dataset and the Hammer dataset were downloaded from the ReCount database (http://bowtie-bio.sourceforge.net/recount) (Frazee et al., 2011). The Pickrell dataset was obtained from lymphoblastoid cell lines derived from 69 unrelated Nigerian individuals as part of the International HapMap project (Pickrell et al., 2010) and contains 69 biological replicates. The Hammer dataset contains four biological replicates in each of two treatment groups: rat L4 dorsal-root-ganglion-treated groups in the presence or absence of induced chronic neuropathic pain (Hammer et al., 2010). The third real dataset was the Arab dataset provided as “arab” in the NBPSeq R package and that includes three biological replicates, where *Arabidopsis* leaves were inoculated with either a defense-eliciting Δ*hrcC* mutant of *Pseudomonas syringae* pv. *tomato* DC3000 or 10 mM MgCl_2_ as a mock-treatment control (Di et al., 2011).

#### Simulated datasets

Simulation studies represent necessary processes for investigating the properties associated with certain statistical methods, given that the “true” DEGs are known in simulated data. An ideal simulation would generate data with similar characteristics to those produced in real RNA-seq experiments. Therefore, similar to Landau and Liu (2013), we generated three independent simulated datasets using a DP model based on three real datasets, respectively. The simulation processes were repeated 30 times to ensure reasonable precision in parameter estimation. Each simulated dataset contains 10,000 genes, including 2,000 DEGs and 8,000 non-DEGs, two treatment groups, and three replicates for each treatment group.

#### Foxtail dataset

Foxtail millet (*Setaria italica*) is an important cereal crop in northern China, and the whole-genome sequence of Foxtail millet (Yugu-1 cultivar) was published in 2012 (Bennetzen et al., 2012; Zhang et al., 2012). In this study, we used a Foxtail RNA-seq dataset obtained by our own laboratory to compare the performance of DREAMSeq with other methods. This Foxtail dataset includes three biological replicates, in which roots from 1-week-old Foxtail millet seedlings (Yugu-1 cultivar) were treated with or without 1 μM epi-Brassinolide (eBL) for 2 h, followed by total RNA extraction using Trizol reagent (Invitrogen, Carlsbad, CA, Unites States). Extracted total RNA (2 μg per sample) was sequenced on an Illumina HiSeq X-ten platform, and the remaining RNA was used for qRT-PCR validation. The paired-end reads were aligned to the Foxtail millet reference genome (JGIv2.0.34) (Bennetzen et al., 2012; Goodstein et al., 2012) using TopHat (version 2.0.12) (Trapnell et al., 2009; Kim et al., 2013), and gene read counts were obtained using the program htseq-count from the python package HTSeq (version 0.61) (Anders et al., 2015).

### qRT-PCR

First-strand cDNA was synthesized from 1 μg total RNA using Reverse Transcriptase M-MLV (Takara Bio, Otsu, Japan) according to manufacturer instructions. qRT-PCR was performed according to the standard protocol using a Bio-Rad CFX Connect real-time PCR system (Bio-Rad Laboratories, Hercules, CA, Untied States). Primers used are listed in Table S1. The expression of target genes was normalized to Foxtail *Actin*, and the relative expression between treatment and control groups was averaged from three independent experiments, with the *p*-value calculated using a one-sample *t*-test. We defined genes satisfying relative expression >1.5 or < 0.67 and *p* < 0.05 as “true” DEGs.

## Results

### The mean–variance relationship in real datasets

When analyzing the Hammer, Arab, and Foxtail datasets, we found strong relationships between the variances and the means on the log-log scale for the read counts from different real datasets (Figure S1). For convenience of notation and calculation, we used the unit line to represent a Poisson assumption-exhibited equidispersion. The data points on and above that line exhibit non-underdispersion, whereas the data points below that line exhibit underdispersion. Figure S1 shows that 2,606 of 18,635 genes (14.0%) in the Hammer dataset, 2,015 of 26,222 genes (7.7%) in the Arab dataset, and 4,412 of 35,158 genes (12.5%) in the Foxtail dataset were estimated as underdispersed genes. Therefore, there are a considerable proportion of underdispersed genes in the RNA-seq data. Furthermore, we noted that the underdispersed data points mostly distributed at low read-count regions (Figure S1). These results suggested that in addition to non-underdispersion, underdispersion also exists in RNA-seq data and should be properly handled during the RNA-seq data-mining process.

Most RNA-seq analysis methods were developed based on an NB model, which is able to capture both equidispersed and overdispersed data but not underdispersed data. In comparison, a DP model can capture all RNA-seq data (Efron, 1986). Using real Hammer, Arab, and Foxtail datasets, we found that both DP and NB models were able to fit read-count data very well (Figure S2). This suggested that the DP model can be used to mine RNA-seq data.

### Generation of simulated datasets

Wu et al. (2013) reported that using real data-driven simulations provided a better estimate for gene-wise dispersions and improved DEG detection, because the true DE status of each gene is known by controlling the settings (Wu et al., 2013). Therefore, we generated three simulated datasets with mean and dispersion parameters estimated from three real datasets based on a commonly used DP model and denoted these as simPickrell, simHammer, and simArab, respectively. The average number of underdispersed genes in simPickrell, simHammer, and simArab was 1299 (13%), 1935 (19%), and 1432 (14%), respectively. As shown in Figure S3, all simulated datasets were very similar to the corresponding real datasets in terms of distributions of the means and dispersions and relationships between means and dispersions. This indicated that our simulated data closely mimicked the real data.

### Type I error rate

**Figure 1:**
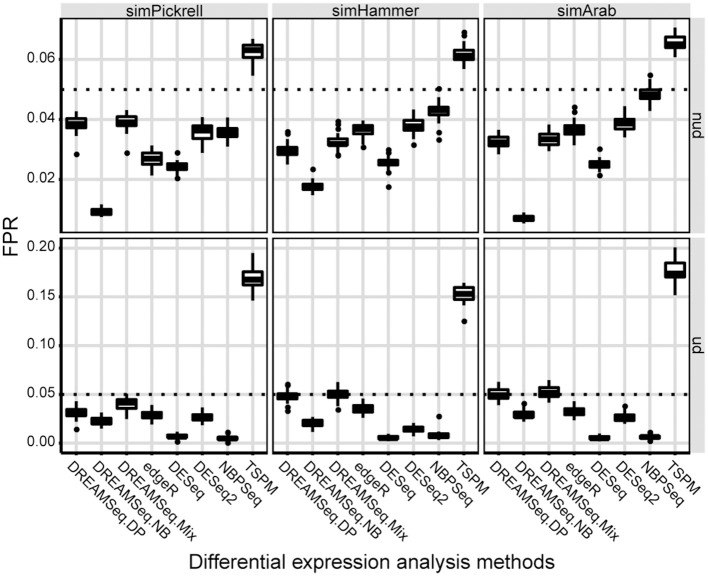
Comparison of type I error rates between different methods. Boxplots show the type I error rates (i.e., FPRs) of different methods, which were calculated over 30 simulations for the simPickrell, simHammer, and simArab datasets under the null hypothesis. The horizontal dotted lines indicate the nominal type I error rate of 0.05 in non-underdispersion and underdispersion scenarios. nud, non-underdispersion; ud, underdispersion.

Using the three simulated datasets, we first evaluated the type I error rates (i.e., FPRs) of the three DREAMSeq methods (DREAMSeq.DP, DREAMSeq.NB, and DREAMSeq.Mix) and five other widely used RNA-seq data-analysis methods (edgeR, DESeq, DESeq2, NBPSeq, and TSPM) under the null hypothesis. We found that except for TSPM, all other methods were able to control type I error rates well in both non-underdispersion and underdispersion situations (Figure 1). In comparison, DESeq was very conservative in term of type I error rate, whereas the abilities of FPR control by both DREAMSeq.NB and NBPSeq clearly varied between non-underdispersion and underdispersion situations. In contrast, the median FPRs of DREAMSeq.DP, DREAMSeq.Mix, edgeR, and DESeq2 were relatively stable and consistently lower than or very close to the nominal type I error rate of 0.05 under all situations.

### Statistical power, ROC, AUC, PRC, and number of DEGs

**Figure 2:**
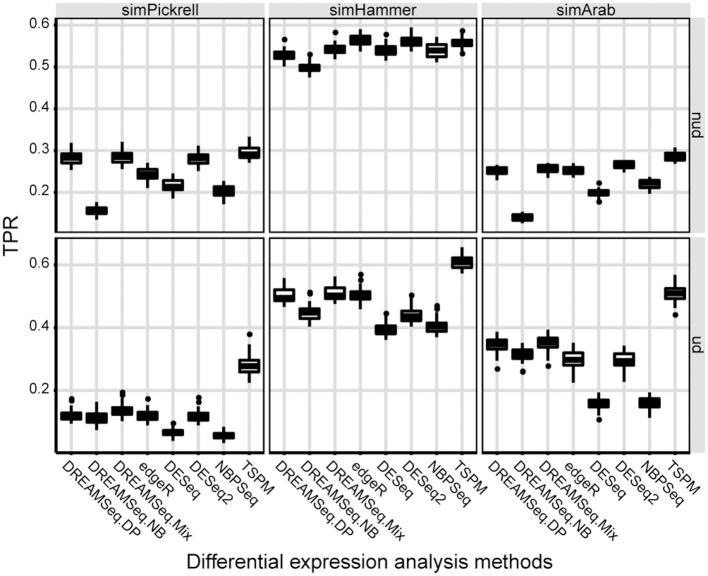
Statistical power comparison between different methods. Boxplots show the statistical powers (i.e., TPRs) of different methods and calculated over 30 simulations for the simPickrell, simHammer, and simArab datasets under the alternative hypothesis in non-underdispersion and underdispersion scenarios. nud, non-underdispersion; ud, underdispersion.

We then evaluated the statistical powers (i.e., TPRs) of different methods using the simulated datasets under the alternative hypothesis (Figure 2). The results showed that in underdispersion situations, the TPR of DREAMSeq.Mix was slightly higher than that of DREAMSeq.DP, although that of both methods was higher than those of DREAMSeq.NB, edgeR, DESeq, DESeq2, and NBPSeq (Figure 2). In non-underdispersion situations, the TPRs of DREAMSeq.Mix and DREAMSeq.DP were comparable with the other methods. Interestingly, TSPM consistently showed higher TPRs. Given that TSPM also showed higher FPRs in similar situations, it is likely that the TSPM method increased statistical power at the cost of poor FPR control.

The ROC curve was constructed using the TPR to FPR ratio for each method used for DE analysis. Theoretically, the method with the stronger statistical power at identifying DEGs should exhibit a ROC curve with a higher TPR relative to other methods at the same FPR level. Figure S4 shows that NBPSeq and TSPM had lower TPRs when the FPR threshold was ~0.05 in each scenario, whereas the ROC curves of the other methods were very similar. Additionally, we found that ROC curves associated with the simHammer dataset were steeper than those for the simPickrell and simArab datasets, suggesting that the performance of DEG identification by different methods was strongly dependent upon innate data characteristics, such as heterogeneity.

**Figure 3:**
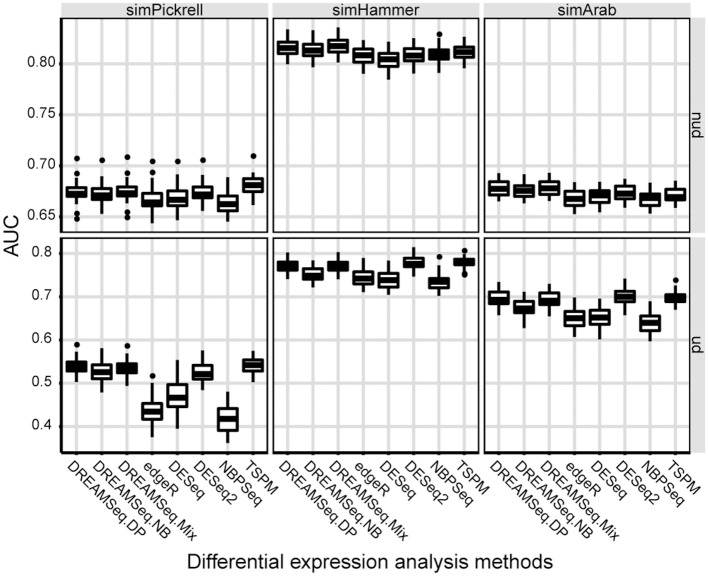
Comparison of AUCs between different methods. Boxplots show the AUCs of different methods and calculated over 30 simulations for the simPickrell, simHammer, and simArab datasets in non-underdispersion and underdispersion scenarios. nud, non-underdispersion; ud, underdispersion.

AUC is a relative measure of the quality of a DEG test, where a higher AUC indicates relatively better performance. To quantify the performances of different methods in detecting DEGs, AUCs of different methods were calculated. The result showed that the AUCs of DREAMSeq.DP and DREAMSeq.Mix were higher than those of DREAMSeq.NB, edgeR, DESeq, DESeq2, and NBPSeq in most of the situations, except slightly lower than DESeq2 when analyzing simHammer and simArab underdispersed data (Figure 3). Together with the above FPR, TPR, and ROC results, these findings clearly demonstrated that both DREAMSeq.DP and DREAMSeq.Mix were able to control type I error rates well while maintaining a relatively higher statistical power in detecting DEGs.

PRC curve shows the precision for corresponding recall (TPR). Similar to the ROC curve, the PRC curve is also an important performance indicator used to evaluate different methods at identifying DEGs. Figure S5 shows that all methods, except TSPM, had higher precision over the entire range of recall rates, regardless of dataset or dispersion. Additionally, we found that all methods exhibited their best predictive performance using the simHammer dataset, but did not predict very accurately using the simPickrell dataset in an underdispersion situation, which might also be related to the dataset itself.

**Figure 4:**
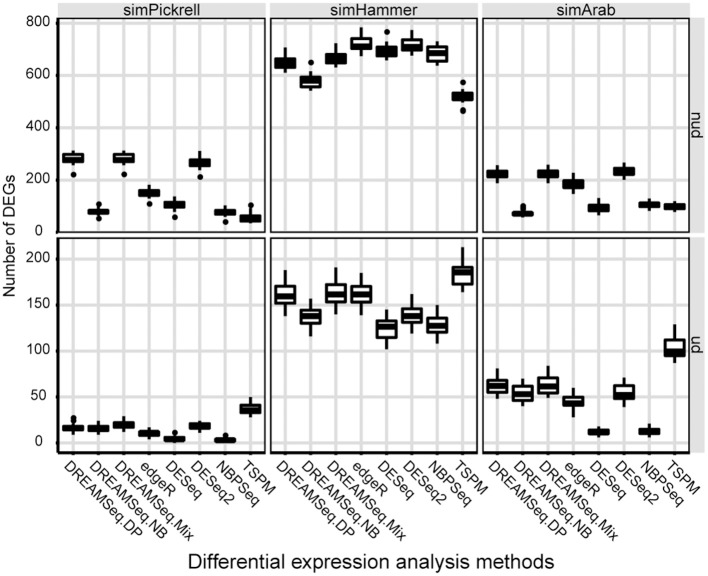
Comparison of the number of DEGs identified by different methods. Boxplots show the number of DEGs identified by different methods and calculated over 30 simulations for the simPickrell, simHammer, and simArab datasets in non-underdispersion and underdispersion scenarios. nud, non-underdispersion; ud, underdispersion.

We also compared the identified DEG numbers of different methods, with the results showing that both DREAMSeq.DP and DREAMSeq.Mix generally detected a larger number of DEGs (except in the case of simHammer non-underdispersed data) than the other methods (except for TSPM, which displayed poor FDR control) when analyzing non-underdispersed or underdispersed data from three simulated datasets, respectively, (Figure 4).

### Analysis of the foxtail dataset

**Figure 5:**
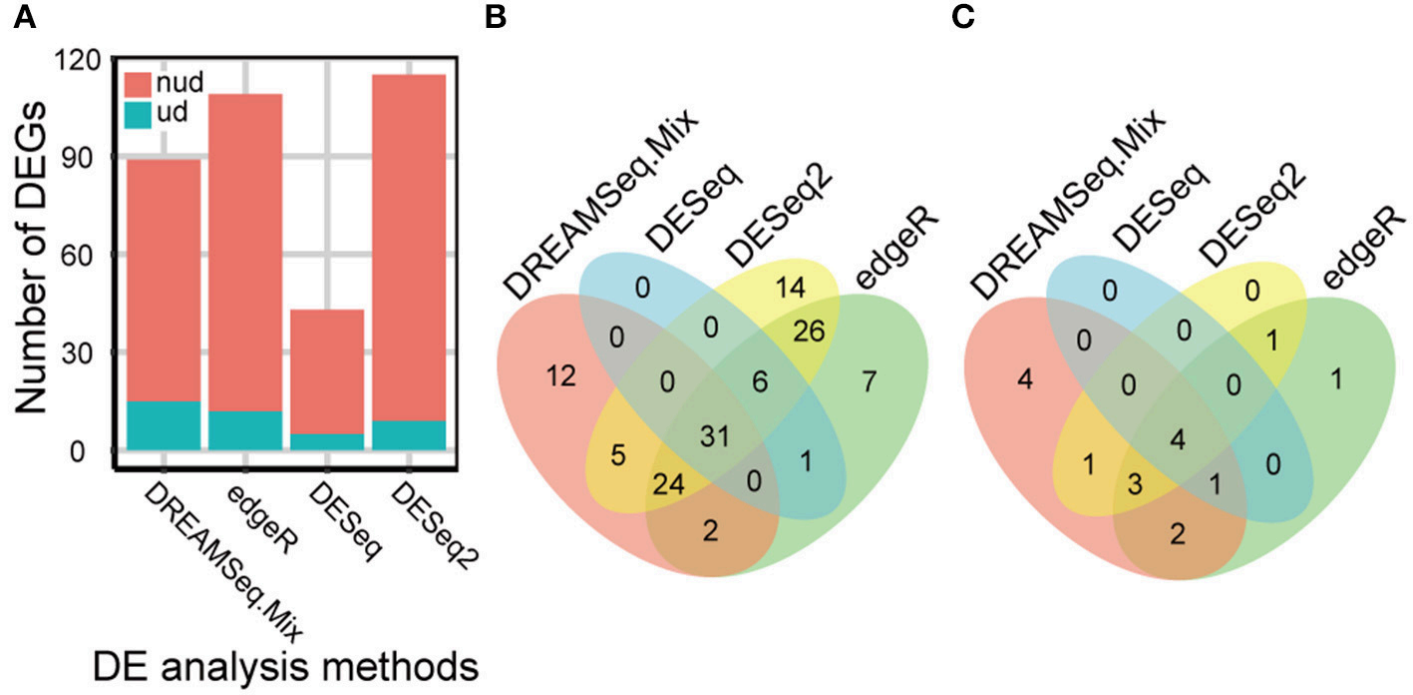
eBL-regulated Foxtail millet-root DEGs identified by different methods. **(A)** Bar plot showing the number of eBL-regulated DEGs identified by DREAMSeq.Mix, edgeR, DESeq, and DESeq2. **(B,C)** Venn diagrams showing the overlap among the collections of eBL-regulated DEGs identified by DREAMSeq.Mix, edgeR, DESeq, and DESeq2 in non-underdispersion **(B)** and underdispersion **(C)** scenarios. nud, non-underdispersion; ud, underdispersion.

Our comprehensive evaluations showed that edgeR, DESeq, DESeq2, and DREAMSeq.Mix generally performed better as analyzing different simulated RNA-seq datasets; therefore, these methods were chosen to test their abilities to detect DEGs, especially underdispersed DEGs, using a real Foxtail dataset. A total of 128 non-underdispersed and 17 underdispersed DEGs were identified by at least one of the four methods (Figure 5 and Tables S2–S5). Overall, the number of DEGs identified by DREAMSeq.Mix was much higher than that by DESeq but lower than that by edgeR and DESeq2 (Figure 5A). However, DREAMSeq.Mix identified 15 underdispersed DEGs, whereas edgeR identified 12, and DESeq2 identified 9 underdispersed DEGs. We defined DEGs detected only by one method as unique DEGs. Notably, DREAMSeq.Mix detected the highest number of unique DEGs in underdispersion scenarios, whereas DESeq did not identify any unique DEGs in either non-underdispersion or underdispersion scenarios (Figures 5B,C). Consistent with previous reports (Seyednasrollah et al., 2013; Tang et al., 2015), all of the DEGs found by DESeq were also found by edgeR (Figures 5B,C), possibly because these two methods use the same statistical model (i.e., the NB model) and hypothesis testing procedure (i.e., the Robinson and Smyth exact test) (Robinson and Smyth, 2008; Anders and Huber, 2010; Robinson et al., 2010). The presence of various unique DEGs also suggested the advantage of using more than one method to analyze the same RNA-seq data in order to allow maximum discovery of DEGs.

**Figure 6:**
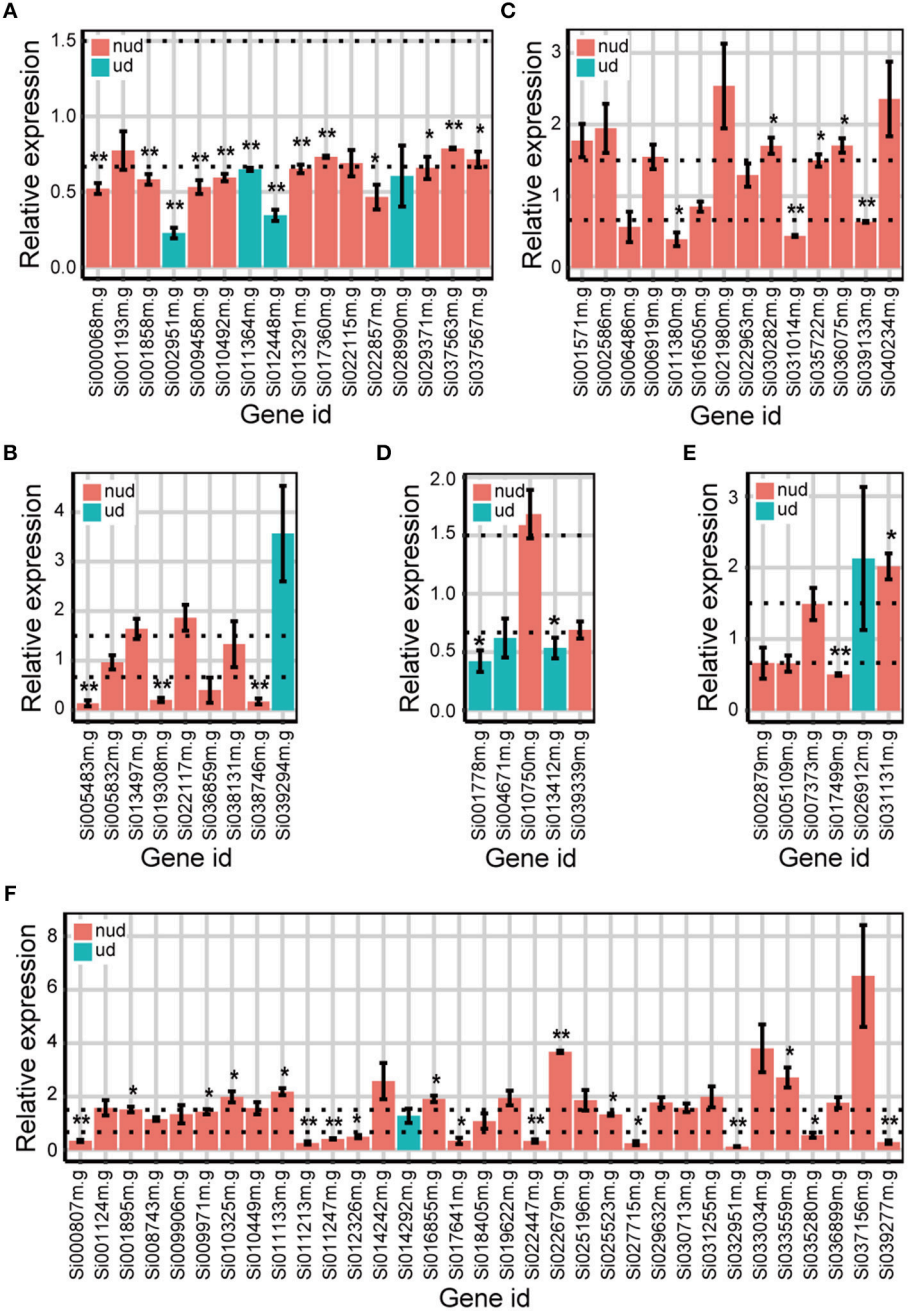
qRT-PCR validation of the expression of eBL-regulated Foxtail DEGs detected by different methods. Bar plots show the relative expression of DEGs detected only by DREAMSeq.Mix **(A)**, edgeR **(B)**, and DESeq2 **(C)** or identified by DREAMSeq.Mix and edgeR **(D)**, DREAMSeq.Mix and DESeq2 **(E)**, or edgeR and DESeq2 **(F)**, respectively, in eBL-treated Foxtail millet roots. The relative expression levels were normalized to the Foxtail millet *Actin* gene. Data represent the mean ± SE of three independent experiments. *P*-values were calculated using a one-sample *t*-test. ^*^P < 0.05; ^**^P < 0.01. The horizontal dotted lines indicate relative expression of 1.5 or 0.67. nud, non-underdispersion; ud, underdispersion.

We then used qRT-PCR to validate whether the DEGs identified from the Foxtail dataset were “true” DEGs. Because DEGs identified by DESeq were also identified by edgeR, the unique DEGs identified by either edgeR, DESeq2, or DREAMSeq.Mix and the common DEGs identified simultaneously by any two methods were chosen for qRT-PCR analysis (Figure 6). The results showed that most of the DEGs chosen for validation exhibited similar upregulation or downregulation patterns as those shown from RNA-seq data analysis. For non-underdispersed DEGs, qRT-PCR results verified that 9 of 19 DEGs (47.4%) identified by DREAMSeq.Mix, 19 of 42 DEGs (45.2%) identified by edgeR, and 23 of 51 DEGs (45.1%) identified by DESeq2 were significantly upregulated or downregulated by eBL treatment by at least 1.5-fold. Notably, for underdispersed DEGs, 5 of 8 (62.5%) DEGs identified by DREAMSeq.Mix were validated as “true” DEGs. By contrast, only 2 of 5 (40.0%) DEGs identified by edgeR and no DEGs identified by DESeq2 were validated as “true” DEGs. These qRT-PCR results demonstrated that for non-underdispersed data, the number of DEGs identified by DREAMSeq.Mix was lower than those by edgeR and DESeq2, but the accuracy was slightly higher; however, for underdispersed data, DREAMSeq.Mix exhibited both a higher number of identified DEGs and better accuracy than the other two methods, demonstrating DREAMSeq.Mix as a powerful RNA-seq data-analysis method, especially for situations involving underdispersed data.

## Discussion

RNA-seq is an increasingly popular method used to analyze global changes in gene expression during certain biological processes. Identifying DEGs is a key step in mining RNA-seq data and important for downstream biological analyses, such as cluster analysis, PCA analysis, GO analysis, and Kyoto Encyclopedia of Genes and Genomes enrichment analysis. When analyzing RNA-seq data, most current methods focus on non-underdispersed data, with less attention given to underdispersed data. In this study, we observed that RNA-seq data also includes underdispersion characteristics. Additionally, Low et al. (2017) found that as the RNA-seq coverage increases, underdispersion becomes increasingly obvious. With the development of sequencing technology, the read length and RNA-seq coverage have increased significantly. Therefore, to take full advantage of RNA-seq data, it is important to explore both non-underdispersed and underdispersed data. However, most widely used DE-analysis methods, such as DESeq and edgeR, are based on the NB model. Due to the limitations of this model, underdispersed data are often overestimated, leading to conservative results in the determination of DEGs. In comparison, the DP model is capable of capturing not only non-underdispersion but also underdispersion. Considering the potential advantages of these two models, we developed a novel RNA-seq data-mining method (DREAMSeq.Mix) that combines the DP and NB models.

Using simulated datasets generated from three real RNA-seq experiments, we compared the performance of DREAMSeq.Mix at detecting DEGs with five other commonly used RNA-seq data-analysis methods. To provide a more comprehensive conclusion, we also added DREAMSeq.DP and DREAMSeq.NB methods, which were developed using only a DP model or an NB model, respectively, into the comparison. We found that DESeq, NBPSeq, and DREAMSeq.NB were often conservative, whereas TSPM, edgeR, and DESeq2 were more liberal in detecting DEGs. The poor performance of TSPM in our study might be due to the limited number of replicates in the RNA-seq datasets used (Auer and Doerge, 2011; Kvam et al., 2012; Soneson and Delorenzi, 2013). In comparison, DREAMSeq.DP and DREAMSeq.Mix often outperformed the other methods in terms of TPR, AUC, and the number of DEGs detected (Figures 2–4). The following reasons suggest that DREAMSeq.Mix provided unique and important outcomes more advantageous than current RNA-seq data-mining methods.

First, DREAMSeq incorporates a more flexible DP model to fit highly complex and variable RNA-seq data. The dispersion parameter of the DP model is not subject to the same restrictions as the NB model when it is estimated in underdispersion situations. As a result, logarithmic dispersion estimated using the DP model (Figure S3) showed a better normality than that acquired using the NB model (Figure 1 in Landau and Liu, 2013). This demonstrated that the DP model was able to accurately fit a widely range of read-count data without artificial intervention in RNA-seq data analysis. Therefore, DREAMSeq.DP and DREAMSeq.Mix often outperformed the other methods, especially in underdispersion situations, in simulation studies. Moreover, in terms of identifying the “true” underdispersed DEGs, DREAMSeq.Mix outperformed edgeR, DESeq, and DESeq2 according to qRT-PCR validation.

Second, DREAMSeq incorporates strategies, such as MoMs, MLE, and EB, which are used in the edgeR, DESeq, DSS, and DESeq2 methods, to obtain reliable dispersion estimation. Importantly, to avoid bias, DREAMSeq used a “window scan” approach to estimate dispersion and enhance DREAMSeq's robustness in analyzing a wider range of RNA-seq data. This enabled all DREAMSeq approaches maintain a higher AUC across different simulated datasets in either non-underdispersion or underdispersion scenarios.

Third, in multiple scenarios, DREAMSeq.Mix performed slightly better than DREAMSeq.DP, although the difference was small. This indicated that the efficiency and robustness of DREAMSeq.Mix was improved by taking full potential of the advantages of the DP and NB models to fit RNA-seq data.

Recently, single-cell RNA-seq (scRNA-seq) has rapidly become a powerful tool for analyzing gene-expression heterogeneity at the individual cell level and been widely applied to diverse fields of biological research, including stem cell differentiation, embryogenesis, and whole-tissue analysis (Saliba et al., 2014). However, scRNA-seq data displays typical features of bimodality (the NB model cannot capture bimodality) (Vu et al., 2016), making such data less efficient for mining using common RNA-seq data-analysis methods. Additionally, Choo-Wosoba et al. (2016) reported that genomic next-generation sequencing data also involves underdispersion. The increased accuracy and robustness displayed in finding “true” DEGs with higher confidence and its better performance at exploring underdispersed data make DREAMSeq a potentially valuable tool for mining sequencing data generated from many other high-throughput platforms, such as scRNA-seq and genomic sequencing.

During our analysis, we found that none of the eight tested methods consistently outperformed other methods under all situations, because different methods are capable of identifying specific groups of DEGs. Although some DEGs can be identified by all methods, the existence of unique DEGs suggested that different methods exhibited specific preferences during DEG detection. Additionally, our study showed that the same method sometimes displayed a wide range of performance variability when analyzing different datasets. It is likely that the intrinsic characteristics of the RNA-seq data determine the appropriateness of one method for data analysis over others. Therefore, to ensure maximum coverage of DEG identification, it is advantageous to use more than one method to analyze the same RNA-seq data. Based on our comparison studies, we recommend that using a combination of edgeR, DESeq2, and DREAMSeq.Mix for RNA-seq data analysis to potentially ensure the maximum retrieval of true DEGs in both non-underdispersion and underdispersion situations.

## Conclusions

Previous studies reported both equidispersion and overdispersion as important characteristics of RNA-seq data. In this study, we showed that underdispersion also exists in RNA-seq data. The NB model widely used in RNA-seq data-mining methods can only capture non-underdispersion but not underdispersion. Here, we presented a DP model capable of capturing not only non-underdispersion but also underdispersion. Given the potential advantages of the two models, we developed a novel RNA-seq data-mining method (DREAMSeq) that combines both the DP and NB models to ensure its flexibility and robustness for RNA-seq data mining. Additionally, we used a “window scan” approach to estimate dispersion and enhance the reliability of DREAMSeq across a wider range of RNA-seq data. Using simulated datasets generated from three real RNA-seq datasets and an in-house-generated Foxtail dataset, we demonstrated the ability of DREAMSeq to reach a better balance between conservative and liberal tests as compared with other methods. Our findings demonstrated DREAMSeq as a reliable and robust RNA-seq data-analysis method that provides important improvements in the DE analysis of RNA-seq data, especially in underdispersion situations.

## Data availability

DREAMSeq R package (version 1.0, Windows binary release) is available publicly (http://tanglab.hebtu.edu.cn/tanglab/Home/DREAMSeq). This package also contains a real Foxtail dataset obtained by our own laboratory.

## Author contributions

WT and ZG designed the research; ZG wrote the DREAMSeq R package and performed all data analyses; ZZ performed Foxtail RNA-seq and qRT-PCR experiments; and WT and ZG wrote the manuscript.

### Conflict of interest statement

The authors declare that the research was conducted in the absence of any commercial or financial relationships that could be construed as a potential conflict of interest.
