# Supplementary material for: DREAMSeq: An Improved Method for Analyzing Differentially Expressed Genes in RNA-seq Data
Source: Front Genet. 2018 Nov 30;9:588. doi: 10.3389/fgene.2018.00588 (PMC6284200; doi:10.3389/fgene.2018.00588)
Supplement: Supplementary file 1 [file Table_1.DOCX]

**Table S1 Summary of primers used in qRT-PCR experiments**

| **Gene id** | **Primer name** | **Primer sequence (5' to 3')** |
| --- | --- | --- |
| Si002951m.g | Si002951-QF | TCAGGATCGGCTTCATCGC |
|  | Si002951-QR | ACGGCAATCTCGTCCTGTTG |
| Si012448m.g | Si012448-QF | GTGGGAATCGTCCAACGTCA |
|  | Si012448-QR | CGATGCACTCAACGTGGAAC |
| Si013291m.g | Si013291-QF | CTAAGAGGTGATGCTCCACGAG |
|  | Si013291-QR | CAACGCCATCCTTTGTCAGC |
| Si017360m.g | Si017360-QF | AGTCATCAAGGCGGCATCAG |
|  | Si017360-QR | GAACCCGGTTGCTGTAGC |
| Si028990m.g | Si028990-QF | TCACTTCACCACAGAGGGTG |
|  | Si028990-QR | TGGGAAACAGCTCGCCATC |
| Si029371m.g | Si029371-QF | GACAAAGACGGCACAGGGAT |
|  | Si029371-QR | TTGTCATCGTCCATGCCGAG |
| Si005483m.g | Si005483-QF | ACAAGGCCAGGCTCTACATC |
|  | Si005483-QR | TCGATCGAGACCATTTGAGGC |
| Si019308m.g | Si019308-QF | CCAAATGATTCCTGGGCGATC |
|  | Si019308-QR | ATGTGTAGCCTGCGAGGAAG |
| Si022117m.g | Si022117-QF | AAGGCGTACGGCTAGAGTTC |
|  | Si022117-QR | TCTGGCAGTTTACGACGAGC |
| Si036859m.g | Si036859-QF | AGAAAGATGCTACGACCGCAGC |
|  | Si036859-QR | GCTGCGCCAAGACATCATCG |
| Si038131m.g | Si038131-QF | AGAACAAGCCGCCCCTGC |
|  | Si038131-QR | CAATGCGGCACCACTTCGATTC |
| Si038746m.g | Si038746-QF | CATCTCACGGCACTCCTCTG |
|  | Si038746-QR | GTCGGTGTAGTGGGACGAAC |
| Si039294m.g | Si039294-QF | TCTTACTATGCGCAGTCCTCG |
|  | Si039294-QR | TAGAGGTTGTCGTACCCGCA |
| Si001571m.g | Si001571-QF | CAACACGCCATTCCTCTTCG |
|  | Si001571-QR | GAGTACACCATCACGCAGTCC |
| Si002586m.g | Si002586-QF | ATGGGTCCTCCTCCAGCAC |
|  | Si002586-QR | TCAGGGTTCCAGATTGGGTG |
| Si006486m.g | Si006486-QF | TTCCGGTCCAACGGTGAG |
|  | Si006486-QR | AACTCGCTCATCAGGTCGTC |
| Si006919m.g | Si006919-QF | CTTCGTCAATGGCTGCGAC |
|  | Si006919-QR | CAGGAGACGGTGGCGTTAC |
| Si011380m.g | Si011380-QF | CAGGAGGCTGATGGCTGAC |
|  | Si011380-QR | TGACGTAGATGAAGCAGCGAG |
| Si016505m.g | Si016505-QF | GATTTGCAGCAAGCGCCAGC |
|  | Si016505-QR | GGGTCGGATTCGAGATCTTC |
| Si021980m.g | Si021980-QF | ACTCTTCAGCTGAGCACGAG |
|  | Si021980-QR | GATTGGCCGCTAGGTTCCTG |
| Si022963m.g | Si022963-QF | TTTCCACCCAAACTCCCCTG |
|  | Si022963-QR | GCGTGAACTGTATCGGGAAC |
| Si030282m.g | Si030282-QF | TCCCCCACAAGATCGGGTTG |
|  | Si030282-QR | GGTTCTTGACGTCGTTGTCC |
| Si031014m.g | Si031014-QF | CGGGTACTACGACTTCCTGC |
|  | Si031014-QR | ACCTGCTCGATCTCCCACT |
| Si035722m.g | Si035722-QF | CTGGTCAACTACGTCGCCTC |
|  | Si035722-QR | GAGTGCAGCTCGAGGATGAG |
| Si036075m.g | Si036075-QF | CAACTTCACCAGTTCCGTCG |
|  | Si036075-QR | TGCTGGTGGTGATAGCCTGA |
| Si039133m.g | Si039133-QF | TGTCATTGGCATTGGGCTC |
|  | Si039133-QR | TGATTGGTAGGAGTTCGCCAC |
| Si040234m.g | Si040234-QF | CTGCTTCGGTGAGGGATGTG |
|  | Si040234-QR | CTTCTCGACGGCGTCCTTG |
| Si001778m.g | Si001778-QF | CTACGTGCTCATCGCCTACC |
|  | Si001778-QR | CCACGAAGTAGAGCACCTGG |
| Si004671m.g | Si004671-QF | GCTACGAGATGAACGACAAC |
|  | Si004671-QR | GGCTCGCACTCGAACTCC |
| Si000068m.g | Si000068-QF | CTCAGGTTGGGTTAACAAGC |
|  | Si000068-QR | TGGGAATCACCGGGTGAG |
| Si001193m.g | Si001193-QF | ATCGCCAGCCTATGCATGG |
|  | Si001193-QR | TCATCCGTCTTGAGGAGGCT |
| Si001858m.g | Si001858-QF | TGCGCCTATGTGCAAGATTTC |
|  | Si001858-QR | GGCATTGTACTCAGCTAC |
| Si002879m.g | Si002879-QF | AGCCTGCGAGACAAAGCAC |
|  | Si002879-QR | ACCAGAGCGCGTACAATCC |
| Si005109m.g | Si005109-QF | GGTGTTCCGCAAGTTCGAC |
|  | Si005109-QR | AACTCGTCGAGGCTGATG |
| Si007373m.g | Si007373-QF | AGCAACAAGAGGAAGAGG |
|  | Si007373-QR | TACGAGGCGTGGAGGGAC |
| Si009458m.g | Si009458-QF | AGTTCCTGCACGAGGTGAAG |
|  | Si009458-QR | TCTGGAAGTAGCCGAGGGAC |
| Si010492m.g | Si010492-QF | TTCAAAGTGCCTTCGTCC |
|  | Si010492-QR | ATCTCCCTGTCAGACAACTC |
| Si011364m.g | Si011364-QF | ACATCCTTCGCCTACAAGCC |
|  | Si011364-QR | AGTGGATGTTGGTGGGGAAG |
| Si017499m.g | Si017499-QF | ACAGATCACGGGATCATCC |
|  | Si017499-QR | CCCAAGGAGTGTTGATGCTG |
| Si022115m.g | Si022115-QF | CATTGGACAGCGCAATTACC |
|  | Si022115-QR | CCACCAACAAACCAGAGAGC |
| Si022857m.g | Si022857-QF | TCCGCTTCCTCTTTGAGGTG |
|  | Si022857-QR | GGCGCTTGCATAGTAACTG |
| Si026912m.g | Si026912-QF | AAGGAGGGCGAGAGCAAG |
|  | Si026912-QR | AGGTGCCGAAGTCGTCGAC |
| Si031131m.g | Si031131-QF | ACGGTTGGAGCGTATGGTG |
|  | Si031131-QR | TGACGTTCCACGAGTCCAG |
| Si037563m.g | Si037563-QF | CAGCAGAAGGTGGTACTGAG |
|  | Si037563-QR | TGTTCTCCTTCAGATCCGCTG |
| Si037567m.g | Si037567-QF | GCTCTGGAGAAGGTTATTCG |
|  | Si037567-QR | CTCCCATCATAGGCGGATAC |
| Si000807m.g | Si000807-QF | ACTGCACCGCTAGACTCG |
|  | Si000807-QR | GAGAGAGTGGTGTCTCGTGC |
| Si001124m.g | Si001124-QF | CCATGGACCCGTTCGACTAC |
|  | Si001124-QR | AAGCCGATGCACTTCACG |
| Si001895m.g | Si001895-QF | ACCTCCAGCCACCAAGACG |
|  | Si001895-QR | AAGAGGTGGCCGTTGATGG |
| Si008743m.g | Si008743-QF | AGAGCTTGGATACTGCAAGG |
|  | Si008743-QR | TGAACACCTCCGCGACATC |
| Si009906m.g | Si009906-QF | GCGTCATGTACCACCCCTAC |
|  | Si009906-QR | CTGGAACACCGGGTACTTG |
| Si009971m.g | Si009971-QF | GGAGTTCAGAGAGGCGATG |
|  | Si009971-QR | TCGAAAGCTGCGTAGTACG |
| Si010325m.g | Si010325-QF | TCTGAACAGGTGGACTGCAC |
|  | Si010325-QR | AAGCAAAGCATCCCCACCTC |
| Si010449m.g | Si010449-QF | ACAGATGCAGGCCATGACC |
|  | Si010449-QR | CCGTTCTCTTCCCAAAGATG |
| Si011133m.g | Si011133-QF | TGCAATCTCAGGGGTGGAGTC |
|  | Si011133-QR | CGCAGCTCGACCACCTC |
| Si011213m.g | Si011213-QF | TTGGGGAGCTGAAGGTGGTG |
|  | Si011213-QR | GGAGCTTCATGTCCTTTTGG |
| Si011247m.g | Si011247-QF | TTGGAGAGCTGAAGGTGG |
|  | Si011247-QR | TCCTTGTCCCTCACCCCTAC |
| Si012326m.g | Si012326-QF | CTTCCAGTCGTTCATCGAC |
|  | Si012326-QR | CCAAAGTCTAGCCGGTGC |
| Si014242m.g | Si014242-QF | TCTCCTACCGCAGCTTCTAC |
|  | Si014242-QR | TCCTGGCAGTAGTTGTAGC |
| Si014292m.g | Si014292-QF | ACACCATCGCCATCCTCTCC |
|  | Si014292-QR | AACACCTGCGCCTTGGCCTC |
| Si016855m.g | Si016855-QF | TGTTCAGCTTCTGGTATCTGG |
|  | Si016855-QR | CGATGCCATAGTAAAGCACTGT |
| Si017641m.g | Si017641-QF | AGGTGCCTATAACTGAGGATG |
|  | Si017641-QR | AGTGCTGGAAGGAATGGAGC |
| Si018405m.g | Si018405-QF | CAGTTCTATAGCTCGCAGG |
|  | Si018405-QR | ATGGTGGGCGAGCACGAGAG |
| Si019622m.g | Si019622-QF | ACGGAAACACCAACAACG |
|  | Si019622-QR | AGGGTGAGGACCAGCTTG |
| Si022447m.g | Si022447-QF | ACCACATGAGTCAATGCTGC |
|  | Si022447-QR | ACCCATTCTCGACTGGAACAC |
| Si022679m.g | Si022679-QF | TCAAGGGTTGCGACGCTTCC |
|  | Si022679-QR | GAGGATGTCGGCGCAGGAG |
| Si025196m.g | Si025196-QF | TGAAGGTCGGGTTCTACAAGC |
|  | Si025196-QR | GATGAGGATGGAGGAGTCG |
| Si025523m.g | Si025523-QF | ACCAGCAGCAGCAGCATCAG |
|  | Si025523-QR | CAGACGACGCAGGCGAGG |
| Si027715m.g | Si027715-QF | TGCTTCGTCGAGGGTTGTG |
|  | Si027715-QR | GTCGATCACCTCGTAGCTC |
| Si029632m.g | Si029632-QF | GATCAAGGAGCCTGCATGG |
|  | Si029632-QR | GTGCGAAGATGAGACCAATGAG |
| Si030713m.g | Si030713-QF | TCACACGTCCCGGTGTTG |
|  | Si030713-QR | CTCCAGGCCTTGTTGTTGTTG |
| Si031255m.g | Si031255-QF | TGATCCCGACGACCTACCTG |
|  | Si031255-QR | TCTTGCTCCAGCGCCATG |
| Si032951m.g | Si032951-QF | AGCACACGAGGAACGATTGG |
|  | Si032951-QR | ATCTGCTCCTGTGCTCTTG |
| Si033034m.g | Si033034-QF | CTACAAGCACTCGTGCCCTC |
|  | Si033034-QR | GTGGAAGGCCATGCGGATGAG |
| Si033559m.g | Si033559-QF | CACCAAGAAGCTTGCCGTTG |
|  | Si033559-QR | CAGGGGAATCCAATCTCCTTCC |
| Si035280m.g | Si035280-QF | ACGAGAAGGTCAGGAGC |
|  | Si035280-QR | TCTTCATCACCTCCGGGTTC |
| Si036899m.g | Si036899-QF | ACGACAACGGTGGTGCGTGC |
|  | Si036899-QR | CGAATCTTGTAGCAGGAGC |
| Si037156m.g | Si037156-QF | CCTCTACCAACGGATCAAGTG |
|  | Si037156-QR | ATCCAATCCGTGCTGGTGC |
| Si039277m.g | Si039277-QF | TGCCAGGAAAAGGAGAGAGC |
|  | Si039277-QR | GCTCAACAGCTTGATCTGG |
| Si005832m.g | Si005832-QF | ATCGCGTTCAGCAGGCGGTTC |
|  | Si005832-QR | AGCAGGATGTTGCTCGGCTTC |
| Si013497m.g | Si013497-QF | TACCACAGGTTCCTTGAGC |
|  | Si013497-QR | TCTCCAGCATCATTGCGTC |
| Si010750m.g | Si010750-QF | ACGTCCCAGTGCTTGCTCC |
|  | Si010750-QR | AGATCCAGTGGTCATCCCAG |
| Si013412m.g | Si013412-QF | ACGGAAGGTGATGCGCTGC |
|  | Si013412-QR | ACCAAGGTTCCTGACAGTGC |
| Si039339m.g | Si039339-QF | TCTGGCACTGACAAGTCTGTTG |
|  | Si039339-QR | TTCACCTTCCCCCAGAAAG |
| Si010361m.g | Si010361-QF | GACGCACAACAGGTATCGTG |
|  | Si010361-QR | TCAAGTGCCACATAGGCGAG |

Gene Si010361m.g is the Foxtail millet *Actin* gene.
