# Supplementary figures and images for: DREAMSeq: An Improved Method for Analyzing Differentially Expressed Genes in RNA-seq Data
Source: Front Genet. 2018 Nov 30;9:588. doi: 10.3389/fgene.2018.00588 (PMC6284200; doi:10.3389/fgene.2018.00588)

## Slide 1
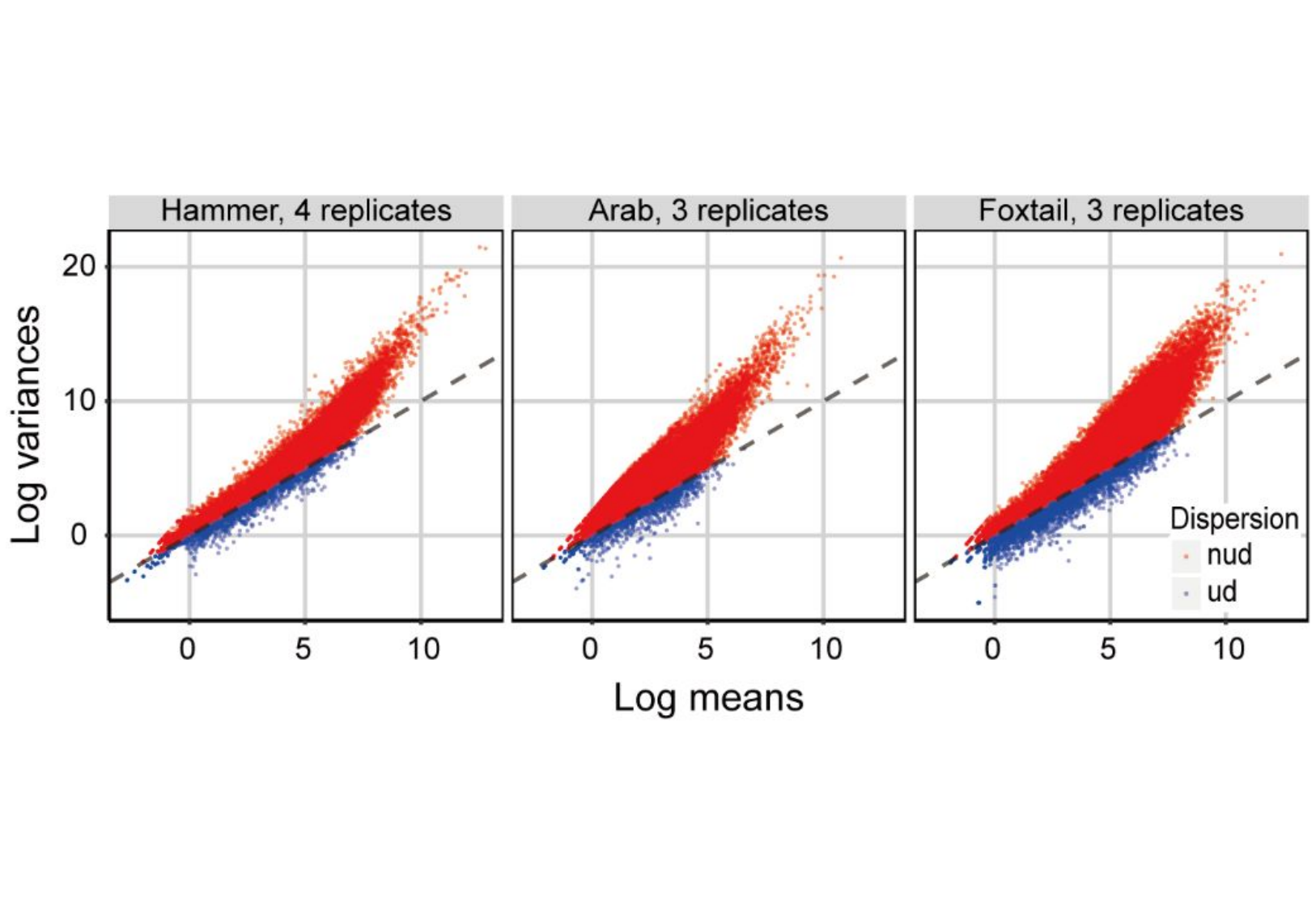

## Slide 2
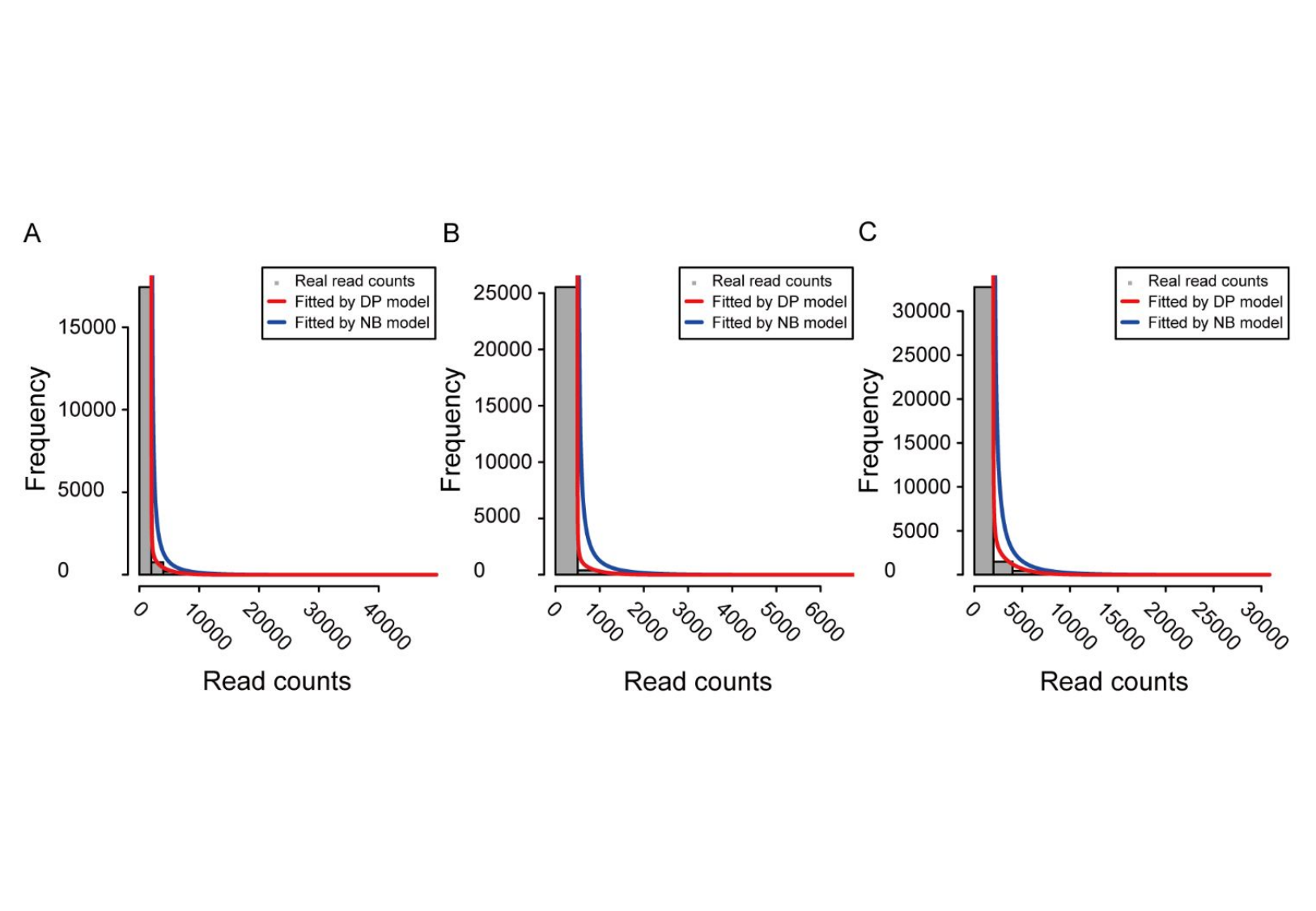

## Slide 3
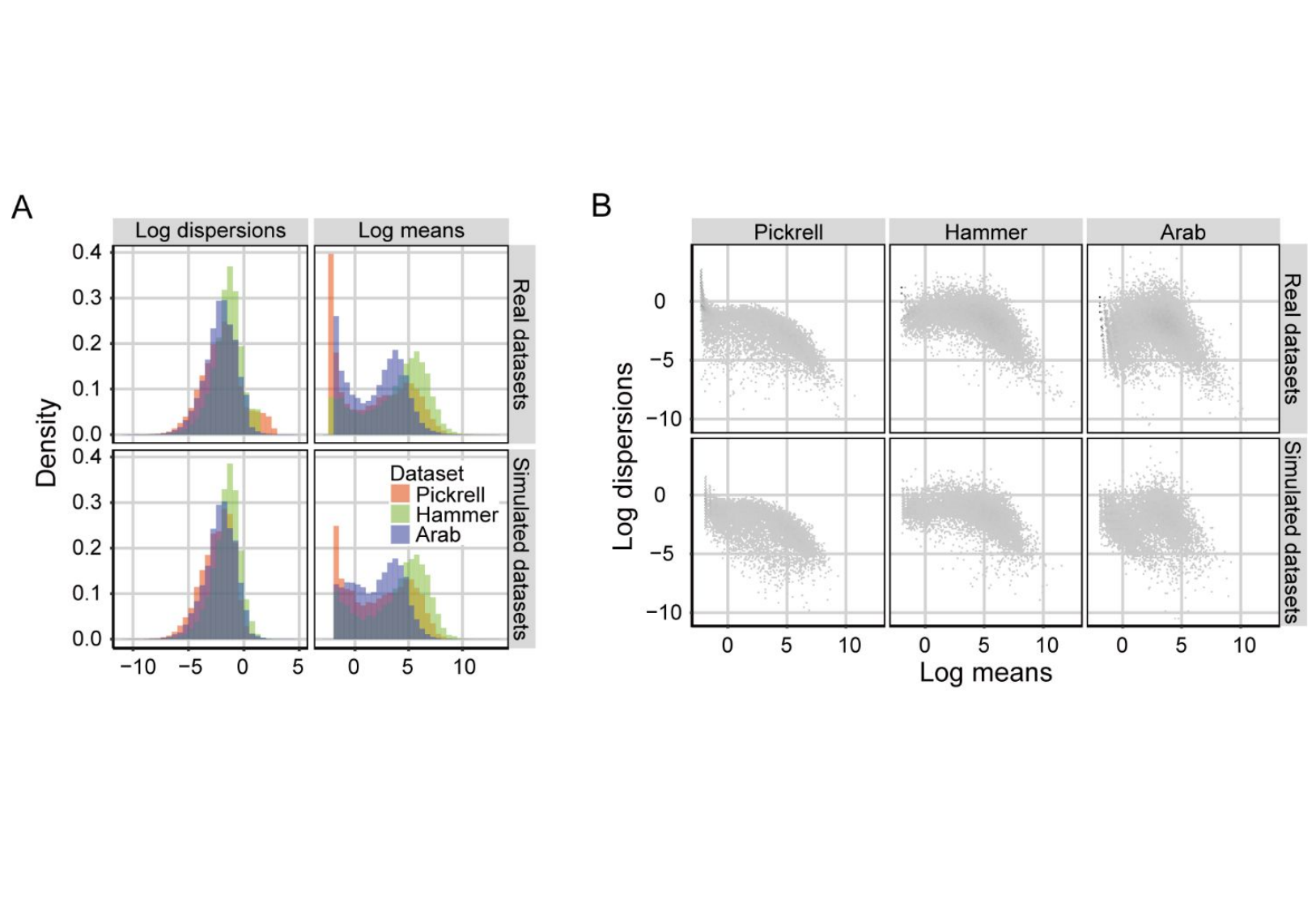

## Slide 4
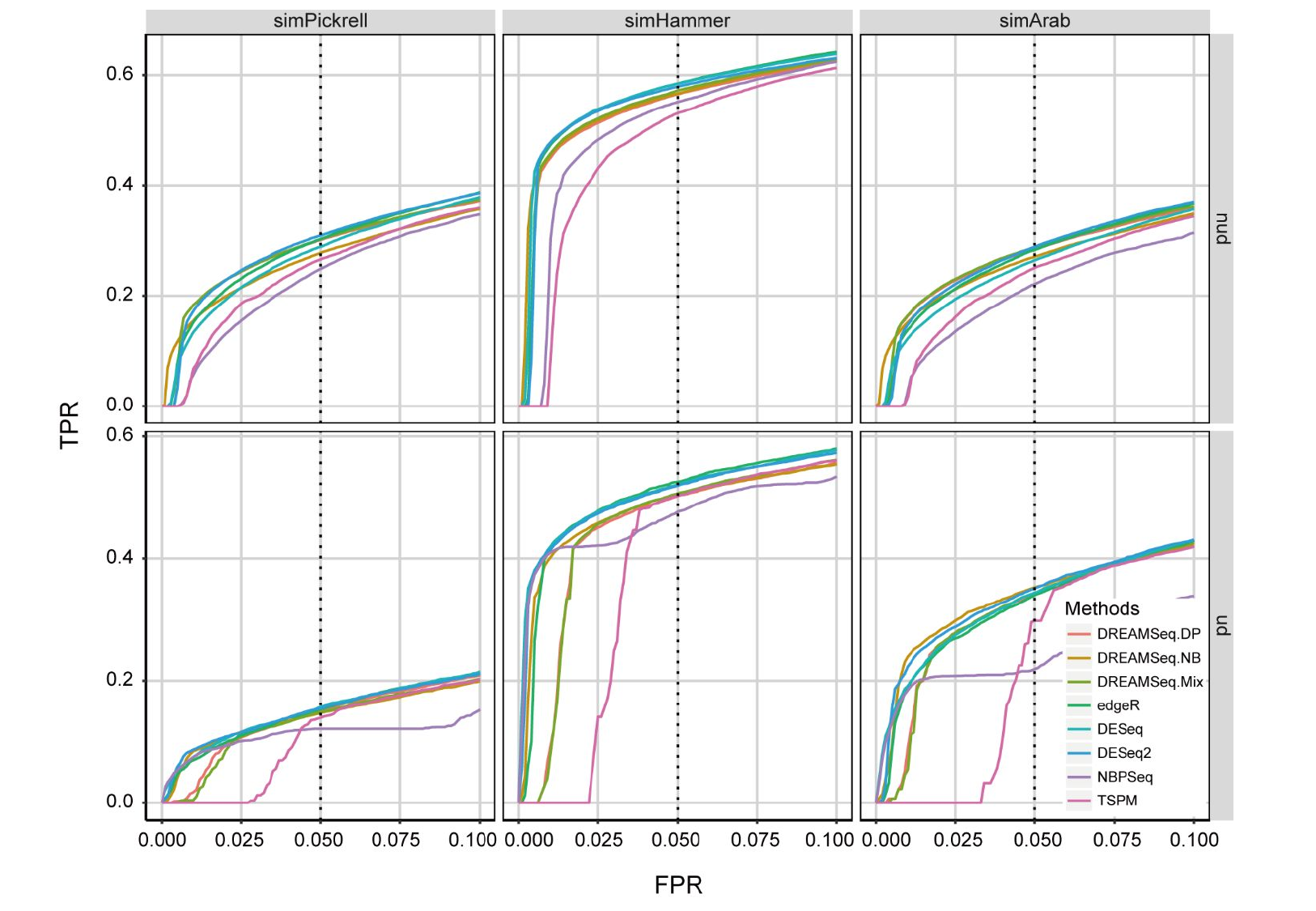

## Slide 5
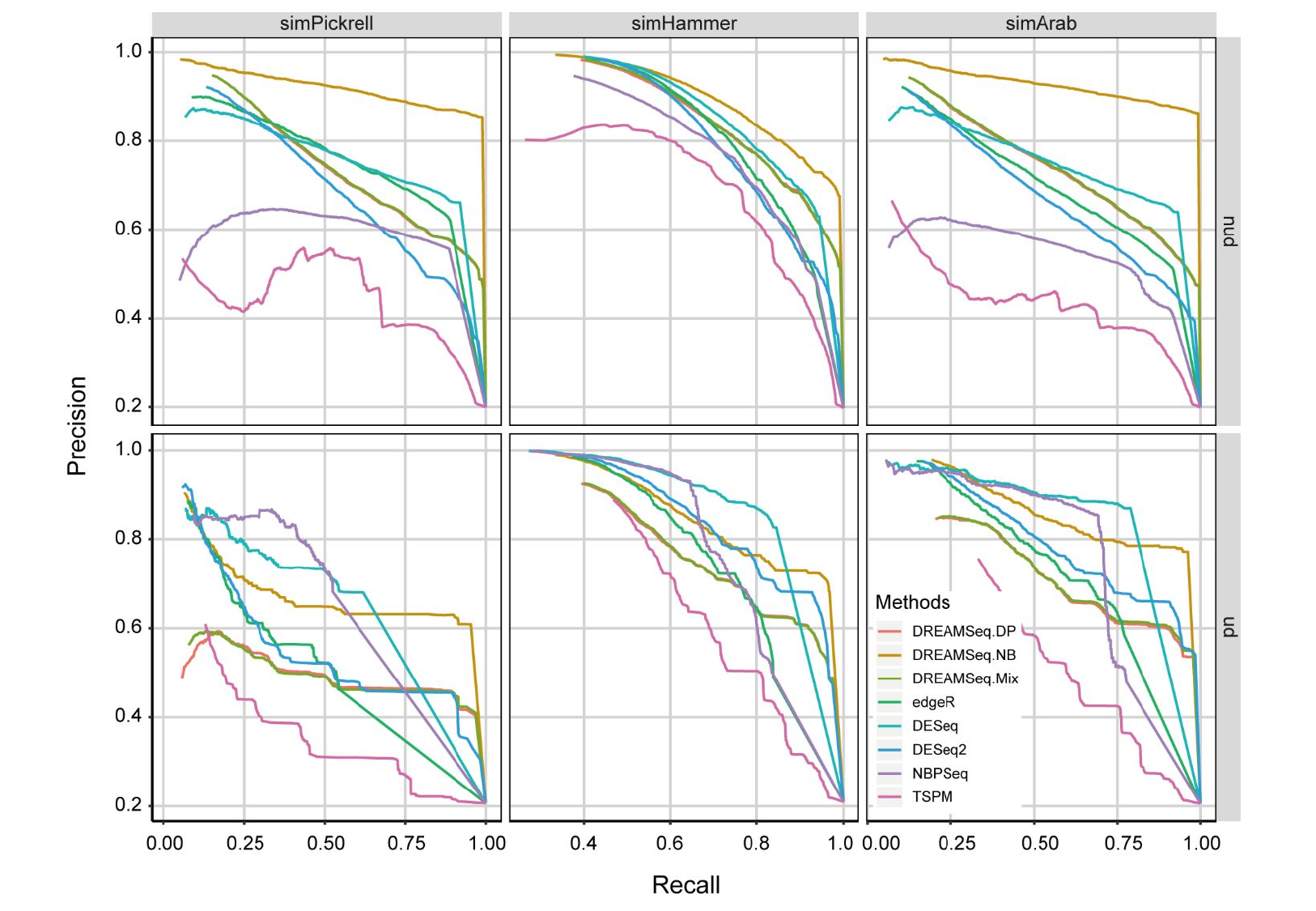

Supplement: Supplementary file 6 [file Presentation_1.PPTX]
